# Effect of Salt Addition Time on the Nutritional Profile of *Thunnus obesus* Head Soup and the Formation of Micro/Nano-Sized Particle Structure

**DOI:** 10.3390/molecules24244447

**Published:** 2019-12-04

**Authors:** Xinyi Fan, Xiaopeng Li, Ningping Tao, Jing Zhang, Mingfu Wang, Xueli Qian, Hong Su, Jian Zhong

**Affiliations:** 1 College of Food Science and Technology, Shanghai Ocean University, Shanghai 201306, China; 15756876511@163.com (X.F.); lee8264@163.com (X.L.); d170202038@shou.st.edu.com (J.Z.); qxl1115281474@163.com (X.Q.); 18616829170@163.com (H.S.); jzhong@shou.edu.cn (J.Z.); 2 Shanghai Engineering Research Center of Aquatic-Product Processing & Preservation, Shanghai 201306, China; 3 Food and Nutritional Science Program, School of Biological Sciences, The University of Hong Kong, Hong Kong, China

**Keywords:** big eye tuna head soup, salt addition time, micro/nanoparticles, Na^+^, Cl^−^

## Abstract

In order to investigate the effects of salt on the nutrients and tastes profiles of big eye tuna head soup, the typical nutrients and taste substances were analyzed. The formation and the morphology of micro/nanoparticles (MNPs) were studied using an inverted optical microscope, and the interactions among components in MNPs were studied using a laser scanning confocal microscope. The results showed that the nutrients were dissolved to the maximum in the soup when salt was added at 150 min of cooking. Comparatively, much smaller MNPs with a more stable bilayer were formed at the same salt addition time. Meanwhile, Cl^−^ was found to permeate throughout the core and Na^+^ bonded with glycosylated molecules, which were dispersed around much smaller MNPs. These results suggested that in addition to promoting the migration of nutrients and taste substances, NaCl also participated in the formation and stability of MNPs in fish head soups.

## 1. Introduction

Tuna, with a world reputation of “marine gold”, is very popular throughout Western and Eastern countries for its abundant omega-3 fatty acids, proteins, and a variety of bioactive substances [1]. The big eye tuna (*Thunnus obesus*) is a major tuna species in the global aquatic product market with a high economic value. However, solid wastes generated from the big eye tuna processing industry, such as viscera, gills, head, bone, and skin, constitute as much as 70% of the original fish [2]. As these aquatic byproducts might waste natural biological resources and cause serious environmental pollution, effective recycle and utilization of these by-products is of critical importance. Soup is a kind of food enjoyed by all people, especially as a good food type for frail and ailing persons due to its good digestibility. Fish soup, including fish head soup, has emerged as a popular food, particularly in Asian culture.

As one of the most common additives used in the food industry, salt (NaCl) plays a major role in the processing, preservation, and sensory acceptability of meat, dairy, baked food, and soup [3]. Notably, the inorganic ions of Na^+^ and Cl^−^ have a prominent effect on the sensory quality of fish. The lack of these ions can cause the disappearance of sweetness and umami, and increase the bitterness significantly [4]. In addition, during soup cooking, salt will not only enhance the dissolution of nutrients and umami substances [5] but also promote emulsification by strengthening the combining capacity among fats, proteins, and polysaccharides [6], leading to the formation of soup micro/nanoparticles (MNPs) as an emerging hot research topic.

As one of the main components of natural food, MNPs have been extensively studied in some types of foods [7,8,9]. Beyond natural components, MNPs could also be endogenously formed during soup preparation. For instance, clam soup was found to contain spherical particles with an average size of 78 nm after being boiled for 1 h [10]. Interestingly, some MNPs in soup showed extra health benefits. As an example, MNPs produced in porcine bone soup after cooking for 3 h could be engulfed by oral macrophages and showed some antioxidant effects [11]. However, the MNPs formed in fish soups and the influence of NaCl on MNPs during the preparation of fish soup have rarely been studied.

In this study, we set up experiments to understand the influence of salt, particularly the timing of the addition of salt to the soup, on nutrient release in big eye tuna head soup, and the formation and stability of MNPs. By instrumental analysis, we investigated the migration of total lipids, eicosapentaenoic acid (EPA) and docosahexaenoic acid (DHA), total sugars, water soluble proteins, free amino acids (FAAs), oligopeptides, and 5′-nucleotides during the cooking process of soup with different times of salt addition, while the formed MNPs were examined by an inverted optical microscopy and laser scanning confocal microscopy (LSCM) with fluorescent dyes. To study the impact of the salt addition time on umami, the contents of 5′-GMP, 5′-IMP, and 5′-AMP were instrumentally analyzed together with umami amino acids. This study will facilitate the utilization of fish heads as soup ingredients and enhance the fish head nutrient release and potential health benefits.

## 2. Results and Discussion

### 2.1. Nutrients Migration

**Figure 1:**
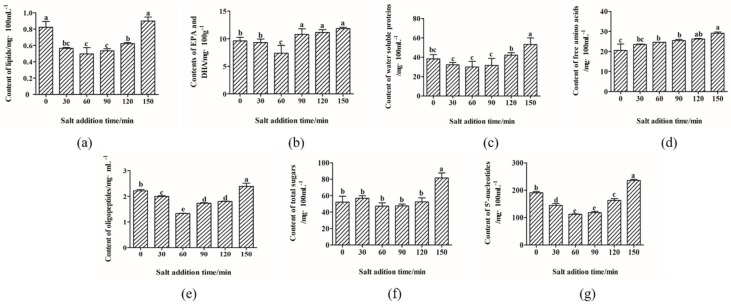
Effects of salt addition time on nutrient migration in big eye tuna head soup. Data points represent means ± standard deviations (*n* = 3). The lowercase letters (**a**–**e**) suggested that the difference of different salt addition time, and the letters are different, suggested that there was significant difference (*p* < 0.05) between data (*p* < 0.05) between data. (**a**) represented total lipids content, (**b**) represented EPA and DHA contents, (**c**) represented water soluble proteins content, (**d**) represented free amino acids content, (**e**) represented oligopeptides content, (**f**) represented total sugars content, and (**g**) represented 5′-nucleotides content. **Notes:** Free amino acids include 17 kinds, such as glutamic acid (Glu), aspartic acid (Asp), and so on. 5′-nucleotides include 5′-guanosine monophosphate (5′-GMP), 5′-inosine monophosphate (5′-IMP), and 5′-adenosine monophosphate (5′-AMP).

The effects of salt addition time on the nutrient migration from big eye tuna head into the soup were evaluated. As shown in Figure 1, the contents of total lipids (Figure 1a), EPA (eicosapentaenoic acid) and DHA (docosahexaenoic acid) (Figure 1b), water soluble proteins (Figure 1c), FAAs (free amino acids) (Figure 1d), oligopeptides (Figure 1e), total sugars (Figure 1f), and 5′-nucleotides (Figure 1g) all reached the highest value when salt was added after the soup was boiled for 150 min. In a previous study, Qian [12] showed that all nutrients reached maximum values after boiling for 150 min. Additionally, further boiling may cause nutrients to undergo degradation and interact with other components. In our study, we found that the contents of most nutrients (e.g., lipids, EPA and DHA, water soluble proteins, oligopeptides, and nucleotides) decreased when the salt addition time was 0, 30, and 60 min during the boiling of the soup, and increased when the addition of salt was delayed at the boiling time of 90, 120, and 150 min. Although it is well-known that salt could increase the osmotic pressure of soup and promote the dissolution of nutrients, due to the complex reaction between NaCl and nutrients, such as promoting the oxidation of lipids [13,14,15] and masking the electrostatic repulsion between proteins after boiling for a longer time [16,17], the time of salt addition had a much complex influence on the migration of nutrients in our observation.

### 2.2. The Inverted Optical Microscopy was Used to Characterize the Morphological Changes in MNPs Dispersed in Big Eye Tuna Head Soups at Different Salt Addition Times

**Figure 2:**
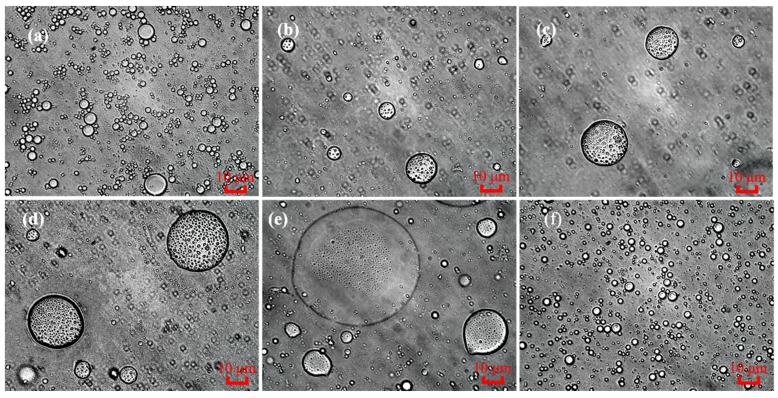
Effects of salt addition time on the formation and stability of MNPs in tuna head soup by inverted optical microscopy. Magnification ×50; Scale bar = 10 μm. The morphology of MNPs in bigeye tuna head soup after boiling for 150 min with the addition of salt at 0, 30, 60, 90, 120, and 150 min. ((**a**) through (**f**), respectively).

Inverted optical microscopy allowed characterization of the morphological changes in MNPs that were dispersed in big eye tuna head soups with different salt addition times (Figure 2). With the extension of the salt addition time, the morphology of MNPs was changed from a regular sphere to irregular. Meanwhile, the aggregation of MNPs was increased and the particle size of MNPs became larger under the same proportional scale. Then, MNPs were converted into stable and regular spheres with a much smaller particles size when the big eye tuna head soup was boiled for 150 min and then salt was added. A previous study showed that MNPs (112 ± 2.4 nm) isolated by size-exclusive chromatography were better than those from original soup as they functioned to alleviate the membrane hyperpolarization induced by hydrogen peroxide free radicals and completely inhibited the aerobic respiration in mitochondria [18].

In the soup sample, when salt was added at the beginning of cooking and boiled for 150 min, it could be observed that there a large number of MNPs with regular sphere shapes but different particle sizes were present, which might be due to some aggregation (Figure 2a). After adding salt prior to cooking, NaCl could increase the binding capacity of proteins to each other and to fats [6], and accelerate the formation of MNPs, which were part of the oil-in-water (O/W) emulsion. Nevertheless, the steric repulsions supplied by NaCl were limited after the longer time cooking, and some aggregation involved the association of the MNPs into flakes without the individual particles being destroyed. Figure 2b–e shows the changes in the morphology of MNPs in big eye tuna head soup after boiling with the addition of salt at 30, 60, 90, and 120 min of cooking, respectively. Compared with adding salt at the beginning of boiling, much larger spherical particles can be seen with the deferred salt addition time. This might be due to the deficiency of steric repulsions during the boiling process before salt was added, eventually leading to the formation of aggregates with larger particle sizes. Furthermore, when salt was added at 30 to 120 min, it can be clearly seen that some unknown substances permeated into the insides of MNPs due to the probable breakdown process of integrated MNPs. As shown in Figure 2f, it is worth noting that a large amount of smaller and stable spherical MNPs were seen to form in the soup compared to Figure 2a. The study of Qian [12] reported that after boiling for 150 min, plenty of stable MNPs in big eye tuna head soup with emulsification characteristics were formed. Then, NaCl added at 150 min means that it did not have enough time to interact with some MNPs.

### 2.3. The Co-Location of Triglyceride and So On in MNPS

**Figure 3:**
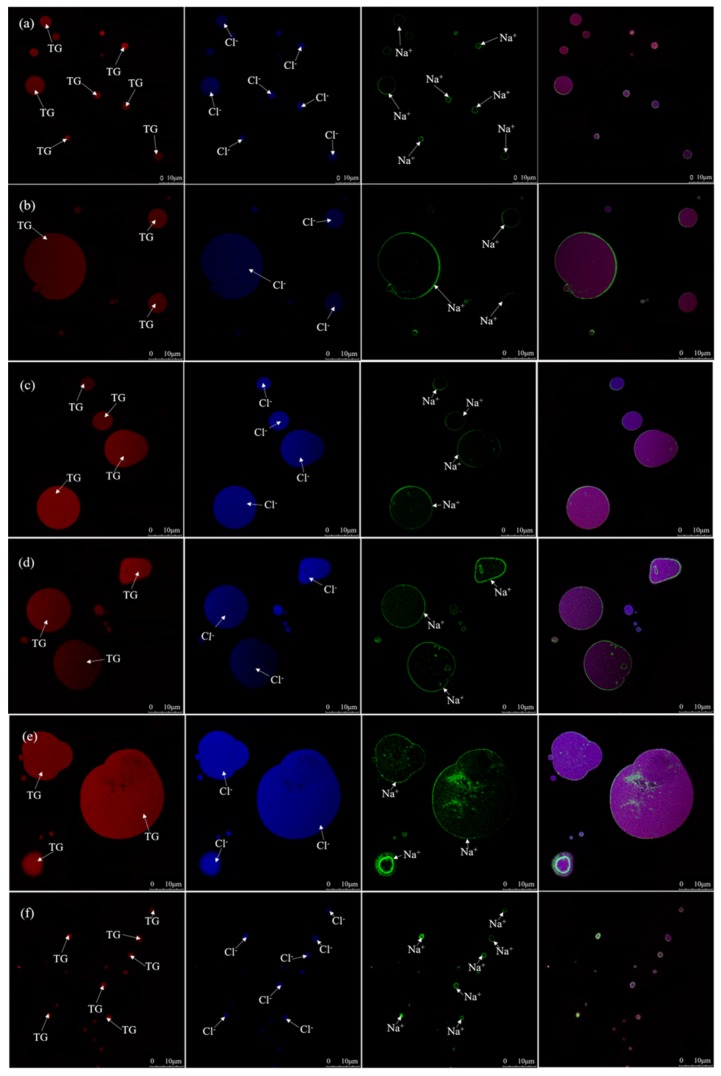
Heterogeneous distribution of triglycerides, Cl^−^ and Na^+^ in MNPs under different salt addition time, co-localized observations performed by the LSCM, used Nile Red (9-diethylamino-5*H*-benzoalpha-phenoxazine-5-one)-stained triglyceride (TG) appears red, the MQAE (N-(Ethoxycarbonylmethyl)-6-methoxyquinolinium bromide)-stained Cl^−^ appears blue, and CoroNa™ Green-stained Na^+^ appears green (for interpretation of the references to the color in this figure legend, the reader is referred to the web version of this article). (**a**) through (**f**) show the co-localization micrographs of triglyceride, Cl^−^, and Na^+^ in MNPs after adding salt at different times (0, 30, 60, 90, 120, and 150 min, respectively) and boiling for 150 min. Images were captured using a ×63 oil immersion objective. The scale bars are indicated in the figures. White arrows correspond to the identified substance.

**Figure 4:**
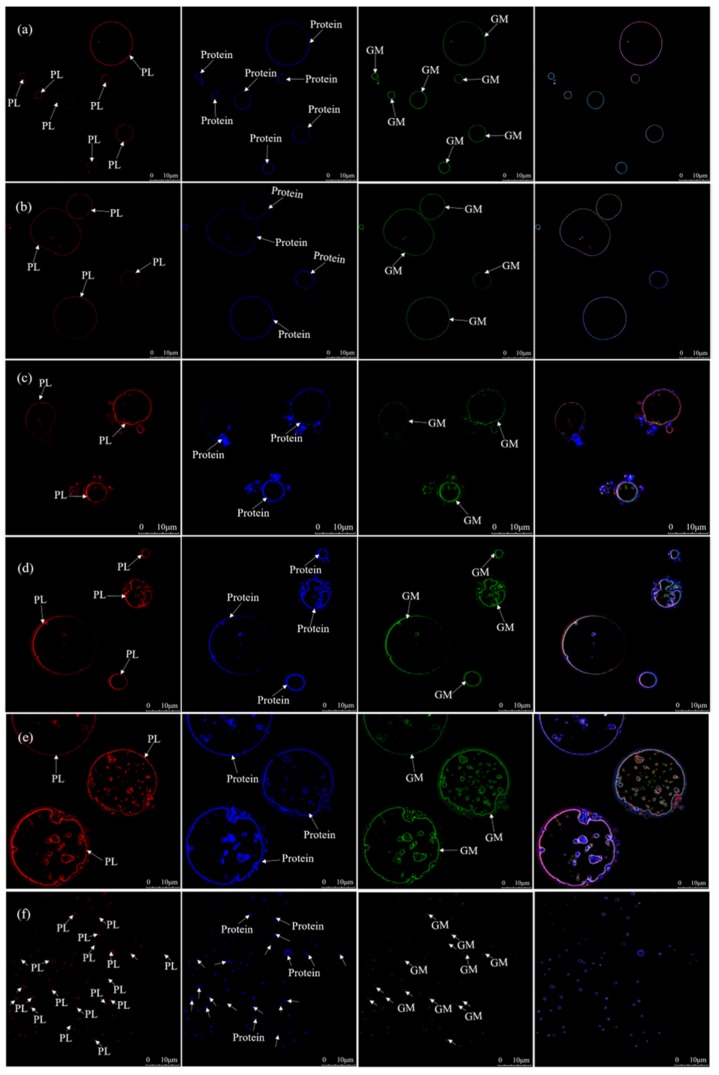
The heterogeneous distribution of polar lipids, proteins, and glycosylated molecules (glycoproteins and glycolipids) in MNPs under different salt addition times, co-localized observations performed by the LSCM, used Rh-DOPE (1,2-dioleoyl-glycero-phosphatidylethanolamine-*N*-(lissamine rhodamine B sulfonyl)) -labeled polar lipids (PLs) (red), Nile blue-labeled protein (blue), and the WGA488 (wheat germ agglutinin Alexa fluor 488)-labeled glycosylated molecules (GMs) (green) (for an interpretation of the references to the color in this figure legend, the reader is referred to the web version of this article). (**a**) to (**f**) respectively show co-localization micrographs of PL, protein, and GM in MNPs after the addition of salt at different times (0, 30, 60, 90, 120, and 150 min, respectively) and boiling for 150 min. Images were captured using a ×63 oil immersion objective. The scale bars are indicated in the figures. White arrows correspond the identified substance.

The effects of the salt addition time on the formation and change of MNPs was further studied using LSCM and fluorescent dyes. Compared with conventional light microscopy, LSCM can co-localize TG, Cl^−^, Na^+^ (Figure 3), PL (Phospholipid), proteins, and GM (Glycosylated Molecules) (Figure 4) in MNPs, respectively.

Figure 3a–f show the co-localization results of TG, Cl^−^, and Na^+^ in MNPs, with the addition of salt at the beginning of the cooking. TGs, the hydrophobic molecules, were exclusively located in the core of MNPs, which is similar to milk fat globules [19]. Cl^−^ was dispersed into the core of spheres. Meanwhile, Na^+^ was situated in the TG spheres’ periphery. It was reported that Na^+^ could be adsorbed on electronegative polysaccharides that were bound to the phospholipids at the periphery of the MNPs [20]. Figure 3b–e show the co-localization of TG, Cl^−^, and Na^+^ of MNPs in big eye tuna head soup with the addition of salt at 30, 60, 90, and 120 min during the cooking process, respectively. The size of the TG spheres gradually increased, which is in accordance with the results observed by inverted optical microscopy. With some MNPs aggregated together, the stable structures of the surface bilayers were destructed, and this phenomenon required an in-depth study. Figure 3f shows the co-localization micrographs of TG, Cl^−^, and Na^+^ in the MNPs formed with the addition of salt at the end point of big eye tuna head soup cooking process. A large number of much smaller MNPs were evenly distributed in soup (Figure 3f), which is consistent with the phenomenon of Figure 2f.

The effects of adding salt into big eye tuna head soup at the beginning of the cooking on the location of PL, proteins, and GM in MNPs were also observed. PL, proteins, and GM were uniformly distributed in the periphery of TG spheres (Figure 4a), which is similar to milk fat globules as well [21]. The co-localization consequence of PL, proteins, and GM at MNPs in big eye tuna head soup with different salt addition times (30, 60, 90, and 120 min, respectively) is shown in Figure 4b–e. Due to the break effect on the surface bilayers of MNPs, MNPs aggregated together with larger particle sizes. At the same time, the interiors of MNPs were permeated with some PL, proteins, and GM, which was mentioned above as an unknown substance. Furthermore, plenty of smaller stable spheres were observed (Figure 4f), which were the same as Figure 3f and Figure 2f.

### 2.4. The Analysis of Umami Taste

The umami substances in fish are mainly composed of umami amino acids, 5′-nucleotides, oligopeptides, and so on. However, the pleasant umami taste is not only caused by the simple accumulation of umami substances but also by the synergistic interactions among them. Yamaguchi et al. [22] investigated the synergy between umami amino acids and 5′-nucleotides, and deduced an empirical formula for the equivalent umami concentration (EUC), which helps to quantify synergistic effects on umami intensity. Bellisle et al. [23] clarified that this synergy is caused by the binding of glutamate (Glu) and 5′-nucleotides to receptor proteins, resulting in a change in its spatial configuration. Schiffman et al. [24] demonstrated an interaction between L-Glu and inorganic cations.

The taste activity values (TAVs) of 5′-IMP, 5′-GMP, and 5′-AMP, which are the flavor nucleotides in aquatic products, were almost higher than 1 (Table 1). Among them, 5′-AMP was the most abundant nucleotide, which is in accordance with the result determined in yellow fin tuna [25]. However, the TAVs of umami amino acids were all less than 1, which means that the umami amino acids were considered as inactive in umami taste [26,27] and may have contributed to the umami taste of the soup through synergistic effects with other umami substances, such as 5′-IMP and 5′-GMP. Additionally, the umami intensity given by the synergistic effect between MSG-like amino acids (Asp and Glu) and 5′-nucleotides (5′-GMP, 5′-IMP, and 5′-AMP) is called monosodium glutamate equivalent (gMSG/100 g). As shown in Table 1, the EUC was the highest (0.54 ± 0.00 gMSG/100 g) when NaCl was added after the big eye tuna head soup was boiled for 150 min, and it showed a significant difference with the other salt addition times (*p* < 0.05). The TAVs of the EUC based on an MSG threshold of 0.03 g/100 mL [28] were all much higher than 1 at all salt addition times, indicating that umami is one of the dominant flavors of big eye tuna head soup.

## 3. Materials and Methods

### 3.1. Materials and Reagents

Big eye tuna heads cut in half (number: 30; length: 28.17 ± 2.10 cm; width 27.30 ± 1.00 cm; weight: 1.75 ± 0.16 kg) were obtained from Dalian Xiang Xiang Food Co., Ltd. (Dalian, China). The fishes were captured from the Pacific Ocean and Indian Ocean, and stored at −30 °C before use. Natural salt (300 g/bag) was obtained from Chongqing Salt Industry Group Co., Ltd. (Chongqing, China). The mixture of 37 fatty acid methyl esters (FAME), C19:0 (purity 99%) and C19:0 FAME, bovine serum albumin (BSA), and anthrone (purity ≥98%) were purchased from Shanghai ANPEL Scientific Instrument Co., Ltd. (Shanghai, China). CoroNa™ Green and MQAE were purchased from Thermo Fisher Scientific Inc (Shanghai, China). Nile Red fluorescent dye was bought from Shanghai Macklin Biochemical Co., Ltd. (Shanghai, China). The fluorescent dyes Rhod-PE and Nile blue were obtained from Avanti Polar Lipids, Inc. (Alabaster, AL, USA). WGA488 was supplied by Biotium (Hayward, CA, USA). Acetone, methanol, chloroform, and hexane of high-performance liquid chromatography grade; folin phenol reagent, hydrochloric acid, perchloric acid (PCA), trichloroacetic acid (TCA), sulfuric acid, nitric acid, silver nitrate standard solution, dimethyl sulfoxide (DMSO), boron trifluoride-methanol (14% in methanol), and other reagents of analytical grade were all purchased from Shanghai ANPEL Scientific Instrument Co., Ltd. (Shanghai, China). Distilled and deionized water was used in all experiments.

### 3.2. Samples Preparation

The frozen big eye tuna heads were defrosted by running water. Then, they were chopped into fragments with the size of 5 × 3 × 2 cm, and subsequently the fragments were washed with water 3 times. After, the head fragments (400 ± 2 g) were fried at 120 °C for about 40 s with 20.0 ± 0.2 g soybean oil (first grade, Qinhuangdao, Hebei, China). Next, the head fragments and water (1:8, *w*:*w*) were added to a pot, and boiled for a total of 150 min at a temperature of 97 ± 2 °C for 30 min, and then 90 ± 2 °C for 120 min. By previous sensory assessment, the suitable salt addition concentration is 0.5%, and 16.00 ± 0.08 g of salt was added into the big eye tuna head soup. Notably, salt was added once at the specific time point (0, 30, 60, 90, 120, and 150 min, respectively) for each sample. The soups were then collected by filtering through a single layer filter and two layers of gauze to remove the residues of meat and bone tissue, and stored at −30 °C.

### 3.3. Lipid Analysis

The method of Folch [29] was used to extract thte total lipids of big eye tuna head soup. In total, 20 mL of soup was mixed with 400 mL of chloroform/methanol (2:1, *v*/*v*), and extracted for 24 h at 4 °C. After filtration of the residue, sodium chloride solution (NaCl, 0.9%, *w*/*w*) was added into the extraction solution and kept at 4 °C for 3 h. The lower chloroform phase was collected and evaporated under vacuum to obtain total lipids. Finally, the obtained total lipids were weighed by an electronic analytical balance (AUY220, Chengdu, China) and stored at −70 °C within nitrogen for future EPA and DHA analysis.

### 3.4. Analysis of EPA and DHA

The lipids of big eye tuna head soup were converted to fatty acid methyl ester derivatives according to the method published by Zhang [30]. Firstly, 0.08 to 0.10 g of lipids were mixed with 100 μL of C19:0 internal standard (10 mol/mL) and 5 mL of methanolic-NaOH (0.5 mol/L). The mixture was then put into a condensing and concentrating device (HWS-24, Shanghai, China), and heated at 100 °C for 10 min. After, 3 mL of boron trifluoride-methanol (14% in methanol) was added to the mixture at 100 °C and shaken for 5 min, followed by the addition of 2 mL of n-hexane and holding at 100 °C for 2 min. Next, 10 mL of saturated NaCl solution was added to the mixture. Finally, the mixture was centrifuged at 5000 r/min for 5 min. After centrifugation, the upper layer was collected using a 2-mL disposable syringe and purified with a nylon syringe filter (13 mm × 0.22 μm) and stored in a 2-mL thread screw neck vial with a septum (32 × 11.6 mm, ANPEL Inc.) and injected into a gas chromatograph for EPA and DHA analysis.

EPA and DHA methyl esters were analyzed by a gas chromatograph (Thermo Fisher Inc., Waltham, MA, USA) equipped with an Agilent SP-2560 capillary column (100 m × 0.25 μm × 0.2 μm) and a flame ionization detector (Thermo Fisher Inc., Waltham, MA, USA). Nitrogen was used as the carrier gas at a flow rate of 1 mL/min. The temperature of the column ramp was: The initial temperature was 70 °C, heated to 140 °C (20 °C/min), held for 1 min; then to 180 °C (4 °C/min), held for 1 min; then to 225 °C (3 °C/min), held for 30 min. The gasifying temperature was 250 °C. The injection volume was 1 μL, with a split ratio of 45:1. EPA and DHA esters were identified by comparison of their retention times with a standard fatty acid methyl ester mixture. The contents of EPA and DHA were calculated using the area ratio of the GC peak between the internal standard C19:0 and the EPA and DHA being tested, expressed as mg/100 g of oil.

### 3.5. Water Soluble Proteins Analysis

The content of water-soluble proteins was measured using Folin-Ciocaileu reagent with BSA as a standard, as described by Lowry [31]. Firstly, 5 mL of big eye tuna head soup was diluted to 100 mL with deionized water, and then 1 mL of the diluted sample was further diluted to 10 mL with deionized water. Then, 1 mL of diluted soup and 5 mL of Folin-Ciocaileu reagent (A solution) were added to a test tube and mixed, and heated at 40 °C for 30 min, followed by the addition of 0.4 mL of Folin-Ciocaileu reagent (B solution), mixed, and heated at 40 °C for 10 min. Finally, the absorbance of the mixture was measured at the absorbance of 500 nm. Calibration was achieved with a BSA standard solution (25, 50, 100, 150, 200, and 250 μg/mL). The results were expressed as mg/100 mL.

### 3.6. The Analysis of Free Amino Acids

The free amino acids (FAAs) were extracted from the big eye tuna head soup according to the method published by Tanimoto [32] with minor modification. In total, 2 mL of soup and 15 mL of 5% trichloroacetic acid (TCA) were added into a centrifuge tube and homogenized. The mixture was ultrasonicated for 5 min and stood for 2 h at 4 °C. Then, the mixture was centrifuged (10,000 rpm, 4 °C) for 10 min, and 5 mL of the supernatant was further collected to a beaker, the pH was adjusted to 2.0 with 6 mol/L NaOH solution and 1 mol/L NaOH solution with the volume brought to 10 mL using deionized water. Finally, the solution was purified with a nylon syringe filter (13 mm × 0.22 μm). The extracted amino acids were analyzed by an automatic amino acid analyzer (L8800; Hitachi Ltd., Tokyo, Japan). The parameters were set as follows: Chromatographic column (4.6 mm × 150 mm, 7 μm), column temperature (50 °C), the flow rate of channel 1 (0.4 mL/min), the flow rate of channel 2 (0.35 mL/min). Mobile phase: The solvents and gradient conditions were described by Zhang [33].

### 3.7. Oligopeptides Analysis

The content of oligopeptides was evaluated by the microscale biuret reaction [34]. Firstly, 2 mL of soup sample and 2 mL of 2% TCA was added to a centrifuge tube, and centrifuged at 4000 r/min for 10 min (Hunan Xiangyi Centrifuge Instrument Co., Ltd., Changsha, China). Then, 1.5 mL of supernatant was removed, and 1.5 mL of 6% NaOH and 0.15 mL of microscale biuret reagent was added and mixed. This was placed at 25 °C for 15 min. Finally, the absorbance of the mixture was measured at 310 nm using an ultraviolet spectrophotometer (L7, Shanghai, China). BSA standard was used as the standard, and the content of oligopeptides was calculated as mg/100 mL.

### 3.8. Total Sugars Analysis

The total sugar content in big eye tuna head soup was analyzed by the anthrone method [35]. In total, 20 mL of soup and 10 mL of 12 mol/L hydrochloric acid were put into a 50-mL flask, and heated in boiling water at 100 °C for 20 min. Next, the mixture was cooled to 25 °C by ice water, filtered, and then brought to 100 mL using distilled water. After that, 4 mL of anthrone reagent were added into 1 mL of sample solution and mixed. Then, the mixture was heated at 100 °C for 10 min, removed, and placed in ice water to cool to 25 °C. The absorbance of each solution was measured at 620 nm (L7, Shanghai, China). Anhydrous glucose was used as the standard, and the content of total sugars was expressed as mg/100 mL.

### 3.9. 5′-Nucleotides Analysis

The extraction of 5′-GMP, 5′-IMP, and 5′-AMP was performed according to the method of Qiu [36] with minor modification. Soup (5 mL) was homogenized in 10 mL of chilled 10% perchloric acid (PCA). After being ultrasonicated for 10 min, the sample was centrifuged in a refrigerated centrifuge (Hunan Xiangyi Centrifuge Instrument Co., Ltd., Changsha, China) at 10,000 r/min for 15 min, and the supernatant was collected into a beaker. Then, 5 mL of chilled 5% PCA were added in the centrifuge tube and the mixture was re-homogenized and centrifuged (10,000 rpm, 15 min). Then, the supernatant was further collected into the same beaker again and neutralized with 6 mol/L KOH to pH 5.8. Lastly, the neutralized supernatant was transferred to a 50-mL volumetric flask and filled to the mark, and then filtered through a membrane with a 0.22-μm pore size. The extracted solution was injected into a HPLC device (2695e; Waters Ltd., Milford, MA, USA) for analysis of 5′-GMP, 5′-IMP and 5′-AMP with isocratic elution. The chromatographic conditions were as follows: ODS-3 chromatographic column (4.6 mm × 250 mm, 5 μm), column temperature (28 °C), flow rate (1 mL/min), injection volume (10 μL), and detection wavelength (254 nm). Mobile phase: A-20 mmol/L KH_2_PO_4_ and 20 mmol/L K_2_HPO_4_ (*v*/*v* 1:1) solutions were mixed and adjusted to pH 5.8 with phosphoric acid, B-methanol, and the ratio of mobile phase A and B was 95:5 (*v*/*v*).

### 3.10. Microstructural Analysis

In order to remove the insoluble impurity and obtain the samples used for the microstructural analysis, the soup was centrifuged (10,000 rpm, 4 °C) for 15 min by a high-speed refrigerated centrifuge (Hunan Xiangyi Centrifuge Instrument Co., Ltd., Changsha, China). Then, 500 μL of sample was placed in a sterilized laser confocal culture dish (35 mm × 20 mm, Thermo Fisher Inc., Waltham, MA, USA) and the morphological characteristics of MNPs in big eye tuna head soups with different times of salt addition (0, 30, 60, 90, 120, and 150 min) were observed with an inverted optical microscope (Shanghai Mingzi Precision Instrument Co., Ltd., Shanghai, China).

Briefly, Nile Red was prepared at a concentration of 42 μg/mL in acetone and used to stain the triacylglycerols (TG) [37]. MQAE was prepared at a concentration of 5 mg/mL in DMSO and used to stain the Cl^−^ [38]. CoroNa™ Green was prepared at a concentration of 500 μg/mL in DMSO and used to stain the Na^+^ [39]. Rd-DOPE was prepared at a concentration of 1 mg/mL in chloroform and used to label the PL [40]. Nile blue was prepared at a concentration of 210 µg/mL in ethyl alcohol and used to label the proteins [41]. WGA488 was prepared at a concentration of 1 mg/mL in phosphate saline buffer and used to label the GM [42]. Then, 100 μL of the Nile Red solution, 40 μL of MQAE solution, and 40 μL of CoroNa™ Green solution were added into 1 mL of soup sample to stained TG, Cl^−^, and Na^+^. In total, 20 μL of Rd-DOPE solution, 10 μL of Nile blue solution, and 10 μL of WGA488 solution were added into 1 mL of soup sample to stained PL, protein, and GM.

The stained samples were kept in the dark for 30 min at 25 °C. Afterwards, the stained sample (10 µL) was added onto a glass slide, slowly mixed with 10 µL of melted agarose, and then slightly put on the cover slip. Finally, the prepared samples were used for microstructural observation.

ALSCM (LSM710 NLO, Zeiss, Oberkochen, Germany) and a ×63 oil immersion objective were used to observe the co-localized TG, Cl^−^, Na^+^, PL, protein, and GM. Fluorescent probes with Nile Red, MQAE, and CoroNa™ Green were excited using an He-Ne laser operated at an excitation wavelength of 543 nm (emission was detected between 565 and 615 nm) and an argon laser operated at an excitation wavelength of 488 nm (emission was detected between 500 and 535 nm), respectively. Additionally, fluorescent probes with Rd-DOPE, Nile blue, and WGA488 were excited using a He-Ne laser operated at an excitation wavelength of 543 nm (emission was detected between 565 and 615 nm), an argon laser operated at an excitation wavelength of 488 nm (emission was detected between 500 and 535 nm), and a diode operated at 633 nm (detected with a long pass filter >650 nm), respectively. The images were processed and analyzed by Zeiss LSM Image Browser off-line software.

### 3.11. Statistical Analysis

All values were from independent triplicates expressed as mean ± standard deviation (SD). Analysis of variance tests were performed using the Statistical Analysis System software (SPSS 20.0, SAS, Cary, NC, USA). All experimental data were compared using Duncan’s multiple range tests (*p* < 0.05) to define the statistical significance. The software of GraphPad Prism (GraphPad Prism 5, GraphPad Software Lab, San Diego, CA, USA) was used to process and produce images.

## 4. Conclusions

The effects of the salt addition time on the release of nutrients and the formation and stability of MNPs in big eye tuna head soup were studied. The results showed that the migration of nutrients and umami substances reached maximum values when salt was added after the soup was cooked for 150 min. With different salt addition times, unstable MNPs with large particle sizes were ultimately observed to be much smaller and stable spheres when salt was added at the end of the cooking (150 min). TG and Cl^−^ were located in the core of the spherical bilayer while PL, protein, GM, and Na^+^ were dispersed in the surrounding bilayer. The results provided evidence that a complex stable MNP system was formed due to the existence of lipids, proteins, sugars, and other components, and the addition time of salt could have an influence on the structures of MNPs, which required an in-depth study on it. This research will provide a theoretical basis in the field of novel self-assembled bioactive structures during soup preparation.

## Figures and Tables

**Table 1:** The influence of the salt addition time on the migration of 5′-nucleotides, umami amino acids, and their TAV and EUC in big eye tuna head soup ^1^.

Umami Compounds		Taste Threshold/mg·100 mL^−1^ [25]	Content/mg·100 mL^−1^						TAV					
			0 min	30 min	60 min	90 min	120 min	150 min	0 min	30 min	60 min	90 min	120 min	150 min
5′-nucleotides	5′-GMP	12.5	42.91 ± 1.66^ab^	42.1 ± 4.57^ab^	39.82 ± 0.34^abc^	34.98 ± 1.45^c^	37.01 ± 2.86^bc^	45.36 ± 1.41^a^	3.430	3.370	3.185	2.798	2.960	3.629
	5′-IMP	25	54.41 ± 1.02^b^	41.25 ± 0.71^c^	33.99 ± 1.08^d^	28.41 ± 1.13^e^	27.7 ± 0.75^e^	66.52 ± 1.62^a^	2.180	1.650	1.359	1.136	1.108	2.661
	5′-AMP	50	93.59 ± 1.17^b^	61.22 ± 1.41^c^	38.13 ± 0.07^e^	54.76 ± 1.37^d^	98.2 ± 2.93^b^	123.74 ± 3.69^a^	1.870	1.220	0.763	1.095	1.964	2.475
umami amino acids	Asp	100	0.44 ± 0.10^d^	0.33 ± 0.00^e^	0.63 ± 0.01^c^	0.86 ± 0.01^a^	0.92 ± 0.02^a^	0.74 ± 0.00^b^	0.004	0.003	0.006	0.009	0.009	0.007
	Glu	30	1.57 ± 0.28^c^	1.65 ± 0.03^bc^	1.68 ± 0.03^bc^	1.90 ± 0.03^ab^	1.99 ± 0.03^a^	1.71 ± 0.01^abc^	0.052	0.055	0.056	0.063	0.066	0.057
	Gly	100	1.14 ± 0.19^a^	1.27 ± 0.01^a^	1.10 ± 0.02^a^	1.21 ± 0.02^a^	1.25 ± 0.01^a^	1.12 ± 0.01^a^	0.011	0.013	0.011	0.012	0.012	0.011
EUC/gMSG·100 g^−1^		0.03	0.4 ± 0.09^b^	0.37 ± 0.01^b^	0.35 ± 0.02^b^	0.43 ± 0.02^b^	0.35 ± 0.01^b^	0.54 ± 0.00^a^	13.240	11.550	11.700	12.440	14.360	17.980

^1^ Results are represented as mean ± standard deviation (*n* ≥ 3). Same superscript lowercase letters in a row indicate no significant difference (*p* > 0.05). Note: The lowercase letters(a–e) suggested that the difference of different salt addition time, and the letters are different, suggested that there was significant difference (*p* < 0.05) between data.
